# Course of body weight before and after the initiation of insulin therapy in type 2 diabetes mellitus: Retrospective inception cohort study (ZODIAC 58)

**DOI:** 10.1002/edm2.212

**Published:** 2020-12-07

**Authors:** Mireille A. Edens, Peter R. van Dijk, Eelko Hak, Henk J. G. Bilo

**Affiliations:** ^1^ Diabetes Research Center Zwolle the Netherlands; ^2^ Epidemiology Unit Department Innovation and Science Isala Hospital Zwolle the Netherlands; ^3^ Department of Endocrinology University of Groningen and University Medical Center Groningen Groningen the Netherlands; ^4^ Groningen Research Institute of Pharmacy University of Groningen Groningen the Netherlands; ^5^ Department of Internal Medicine University of Groningen and University Medical Center Groningen Groningen the Netherlands

**Keywords:** insulin, pharmacoepidemiology, type 2 diabetes, weight management

## Abstract

**Aims:**

The aim of this study was to explore the effect of insulin treatment initiation on weight by taking weight change prior to initiation into account.

**Materials and methods:**

We performed an observational retrospective inception cohort study, concerning Dutch primary care. We identified all patients that initiated insulin treatment (*n *= 7967) and individually matched patients with a reference patient (*n *= 5213 pairs). We obtained estimated mean weight changes in the five years prior to five years post insulin therapy. We applied linear regression analysis on weight change in the first year after insulin therapy (T0 to T+1), with matched group as primary determinant adjusted for pre‐insulin weight change and additional covariates.

**Results:**

Estimated mean weight increased in the five consecutive years prior to insulin therapy (−0.23 kg in year T‐5 to T‐4, 0.01 kg in year T‐4 to T‐3, 0.07 kg in year T‐3 to T‐2, 0.24 kg in year T‐2 to T‐1, and 0.46 kg in year T‐1 to T0) and continued to increase in the first year after, that is T0 to T+1, at a slightly lower rate (0.31 ± 3.9 kg). Pre‐insulin weight change had the highest explained variance and was inversely and independently associated with weight change (p < .001). Starting insulin was associated with weight increase, independent of pre‐insulin weight change (β‐adjusted 1.228, p < .001). Stratification revealed that despite having a more or less similar baseline BMI, patients with substantial weight increase showed higher estimated mean BMI’s followed by weight loss pre‐insulin. In matched references, estimated mean weight changes were negative in all years concerning the study period, indicating consistent weight loss.

**Conclusions:**

Initiation of insulin therapy was independently associated with weight increase; however, overall effect on weight was small and subject to substantial variation. Pre‐insulin weight change is identified as a relatively strong inverse determinant of weight change after insulin initiation.

## Graphical abstract

This paper is the first to specifically include weight changes prior to the initiation of insulin therapy in T2DM treated in primary care, in studying post‐insulin initiation weight change. Remarkable differences in longitudinal courses between patients that initiated insulin and their individually matched references and between post‐insulin initiation weight change strata are demonstrated.

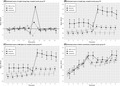

## 1 INTRODUCTION

When dietary measures and oral glucose lowering drugs (OGLDs) fail to sufficiently correct glucose dysregulation, most treatment guidelines for type 2 diabetes (T2DM) advise to start or add either an injectable glucagon‐like peptide‐1 analogue or insulin therapy.^1^ Insulin is widely known and used for its glucose lowering effects, but also is an anabolic hormone affecting lipid and protein metabolism.^2^, ^3^

Several reports claim that insulin therapy is associated with weight gain.^4^ Moreover, the mechanisms behind weight changes after initiation of insulin therapy are multifaceted and not completely understood.^2^ Observational studies on the initiation of insulin therapy in patients with T2DM have shown considerable variations in weight gain between patients,^5^, ^6^, ^7^, ^8^, ^9^, ^10^, ^11^, ^12^, ^13^, ^14^ which raises the question whether and to what part weight gain is attributable to insulin, and whether and to what part weight gain is attributable to other characteristics.

Some differences in weight gain between insulin regimens and types were reported,^6^, ^9^, ^12^, ^13^ but also large standard deviations dwarfing these differences.^9^ Studies have unanimously reported an inverse association of baseline BMI with weight change after initiation.^6^, ^8^, ^10^, ^11^, ^12^, ^13^, ^14^ Results on baseline HbA1c vary from not significantly^7^, ^13^ to significantly^6^, ^8^, ^12^ associated with weight change. Other reported variables include HbA1c change, limited to the first nine months^10^ or the first year^6^ after initiation, and insulin dose and HbA1c at follow‐up.^6^

No strong baseline determinants have been found.^10^, ^13^ The performance of prediction models increased when variables gathered after the decision to start insulin therapy were included.^6^, ^15^ Few studies included weight change prior to the initiation of insulin therapy.^9^, ^16^, ^17^ To the best of our knowledge, there are currently no large observational intervention studies, specifically studying long‐term information on body weight prior to the initiation of insulin therapy in primary care.

We therefore explored the effect of initiation of insulin therapy on body weight by taking weight change prior to initiation into account, using the available data of the Zwolle Outpatient Diabetes project Integrating Available Care (ZODIAC) cohort.

## 2 MATERIALS AND METHODS

### 2.1 Setting and participants

This study is part of the studies in the ZODIAC project, performed in a prospective primary care T2DM patient cohort in the Netherlands, initiated in 1998 for benchmarking purposes.^18^ Exclusion criteria were insufficient cognitive capabilities or a very short life expectancy, based on the judgement of the general practitioners. More than 99.5% of patients also consented with the use of their anonymized data for research purposes. Patients included in the ZODIAC cohort were diagnosed with T2DM and treated in primary care, according to national guidelines, that is the Dutch College of General Practitioner Guideline (Dutch: NHG‐Standaard).^19^ The ZODIAC project was approved by the Ethics Committee of Isala, Zwolle (references 03.0316 and 07.0335).

### 2.2 Study design

Using the available data in the ZODIAC database of the years 1998 to 2014, we performed a retrospective observational intervention study on the effects of the initiation of insulin treatment on body weight, by means of a new user design/ inception cohort.^20^ Figure S1 schematically shows the selection of patients and data for this study. Of all ZODIAC participants, we identified patients that started insulin therapy. Besides extracting data at T0, which is the first registered insulin use signal defined as index time point, we also extracted data of the 5 years prior to T0 and the 5 years after T0.

In addition, a matched reference group of ZODIAC participants who did not start insulin therapy during the study period was selected. Insulin users were individually matched with a referent T2DM patient based on sex, age (±1 year), diabetes duration (±1 year) and BMI‐field (±0.5 kg/m^2^) at T0 in a 1:1 ratio.

### 2.3 Data sources and measurement

A data set of quality indicators on T2DM care was collected annually by general practices. This core data set included patient demographics, laboratory results, medication use, lifestyle and variables collected through physical examination.

HbA1c and lipid profile were determined using standard laboratory procedures. For this study, HbA1c measurement units were aligned to allow proper comparison. HbA1c measured in DCCT/ NGSP units (%) was converted to IFCC units (mmol/mol) using the following formula: HbA1c mmol/mol = (10.93 * HbA1c %) – 23.5.^21^ HbA1c was measured to the nearest mmol/mol. Weight was measured to the nearest kilogram (kg). In the case of a missing BMI‐field, BMI (kg/m^2^) was calculated from weight and height recordings. Lifestyle factors were self‐reported. Physical activity was considered adequate when reported as being performed at least five times a week with a duration of thirty minutes. Clinically unlikely data points were excluded from analysis, as described in Table S1.

T2DM treatment was categorized into four main groups: 1] diet alone, 2] OGLDs, 3] insulin and 4) combination of OGLDs and insulin. Based on Barnett et al^22^ insulin regimens were categorized into five groups: 1] short‐acting insulin and rapid‐acting insulin analogues, 2] premixed types (short‐ and intermediate‐acting), 3] basal insulin (long‐acting), 4] Neutral Protamine Hagedorn (NPH) insulin (intermediate‐acting) and 5] combinations.

### 2.4 Outcomes, exposure and covariates

#### 2.4.1 Outcomes

Primary outcomes were 1] the longitudinal course of weight change concerning the time period T‐5 to T+5, and 2] weight change in the first year after index/ insulin registration (T0 to T+1) specifically. Weight change in kg per year was calculated by subtracting weight measured in consecutive years (new weight – old weight)^23^; hence, a positive value indicates weight gain.

The longitudinal course of estimated means with 95% confidence intervals (95%CIs) of weight change concerning the time period T‐5 to T+5 was visualized, in order to explore changes before and after insulin therapy initiation. Weight change in the first year after index was the primary outcome concerning regression analysis.

#### 2.4.2 Exposure

The exposure of interest was initiation of insulin treatment, which was represented by the insulin subgroup. Non‐exposure was represented by the matched references.

#### 2.4.3 Covariates

Pre‐insulin weight change was studied as main covariate. For this purpose, several measures of weight change concerning the time periods T‐3 to T0, T‐2 to T0 and T‐1 to T0 were studied. Both weight changes in the pre‐insulin time periods (eg T0 minus T‐2) and averaged weight changes of consecutive years within the time periods (eg T‐1 minus T‐2, and T0 minus T‐1, divided by 2) were calculated.

Additional potential covariates included baseline weight and HbA1c,^6^, ^8^, ^12^ (changes in) HbA1c,^6^, ^10^ metformin and sulphonylurea use,^1^ lipid lowering drug use, diuretic drug use,^24^ and physical activity,^25^ and HbA1c at T+1.^6^

### 2.5 Stratified analysis

Longitudinal courses were stratified by weight change category in the first year after index. Weight change was categorized into three categories: 1] substantial weight gain (≥5 kg),^6^ 2] moderate weight gain (1 to < 5 kg) and 3] weight gain less than 1 kg, that is either mild gain, no change or weight loss.

### 2.6 Statistical methods

Analysis sets were as follows: The full analysis set (FAS), containing all patients identified to have started insulin therapy in the study period. The intention‐to‐treat (ITT) analysis set, containing all patients with available weight change data in the first year after index, whether or not they continued insulin therapy.^6^, ^13^ The per‐protocol (PP) analysis set, containing patients with available weight change data that continued insulin therapy at T+1. Patients were included in the PP analysis set whether or not they changed to a different insulin regime. Finally, patients with available weight change data without insulin use at T+1 were indicated as those that used insulin for less than one year (U < 1y).

Categorical data were presented by *n* (%), and quantitative variables were presented by mean with standard deviation (SD) or median (Q1 ‐ Q3) depending on the distribution.

Estimated means with 95%CIs of weight change, weight, BMI and HbA1c were obtained through linear mixed model analyses for repeated measures which allows for extrapolation of missing values.^16^ Weight change, weight, HbA1c and BMI were dependent variables, and time was a fixed factor. Akaike's information criterion was used to select covariance structures.

We performed linear regression analysis to assess the effect of initiation of insulin treatment on weight change, relative to matched references. The dependent variable was weight change (kg) in the first year after index, and matched study group, pre‐insulin weight change and additional covariates were studied as independent variables.

We performed sensitivity analysis concerning weight change in the year prior to index. This was added due to the fact that insulin therapy was initiated in the time period T‐1 to T0, but inherent to a new users design the exact timing of insulin initiation could not be identified.^20^

Analyses were performed using RStudio version 1.1.442, MedCalc version 19.0.5 and SPSS version 26. A 2‐tailed p‐value 0.05 was used to indicate statistical significance.

## 3 RESULTS

### 3.1 Participants

We identified *n *= 7967 patients that started insulin therapy during the study period. None of the patients used insulin before T0. For *n *= 5213 patients that started insulin therapy an individually matched reference patient was available. Table 1 shows patient characteristics for the FAS of both the complete insulin group and the matched groups.

**TABLE 1:** Patient characteristics

	All patients (*n *= 7967)		Matched groups			
			Insulin subgroup (*n *= 5213)		Reference group (*n *= 5213)	
	*N*	Summary statistics	*N*	Summary statistics	*N*	Summary statistics
Demographics						
Age (years)	7966	68.8 (±11.7)	5213	68.4 (±10.7)	5213	68.4 (±10.7)
Sex (men)	7967	3730 (46.8%)	5213	2564 (49.2%)	5213	2564 (49.2%)
Age at diagnosis (years)	7842	58.6 (±11.6)	5213	59.9 (±10.3)	5213	60.0 (±10.3)
Diabetes duration (years)	7842	9 (6–13)	5213	8.4 (±4.3)	5213	8.4 (±4.3)
Medication						
Main treatment group						
Diet	7967	0 (0.0%)	5213	0 (0.0%)	5213	2103 (40.3%)
OGLD		0 (0.0%)		0 (0.0%)		3110 (59.7%)
OGLD and insulin		5542 (69.6%)		3766 (72.2%)		0 (0.0%)
Insulin only		2425 (30.4%)		1447 (27.8%)		0 (0.0%)
Insulin number						
1	7967	7022 (88.1%)	5213	461 (89.4%)	5213	0 (0.0%)
2 or 3		945 (11.9%)		552 (10.6%)		0 (0.0%)
Insulin types (not exclusive)						
Short‐acting	7967	1173 (14.7%)	5213	715 (13.7%)	5213	0 (0.0%)
Premixed		2871 (36.0%)		1574 (30.2%)		0 (0.0%)
Basal		3225 (40.5%)		2390 (45.8%)		0 (0.0%)
NPH		1651 (20.7%)		1089 (20.9%)		0 (0.0%)
OGLD number						
0	7967	2425 (30.4%)	5213	1447 (27.8%)	5213	1738 (33.3%)
1		3439 (43.2%)		2265 (43.4%)		1298 (24.9%)
2 or 3		2103 (26.4%)		1501 (28.8%)		1361 (26.1%)
OGLD types (not exclusive)						
Metformin	7967	4838 (60.7%)	5213	3278 (62.9%)	5213	2598 (49.8%)
Sulphonylureas	7967	2697 (33.9%)	5213	1906 (36.6%)	5213	1711 (32.8%)
Thiazolidinediones	7967	61 (0.8%)	5213	42 (0.8%)	5213	122 (2.3%)
Repaglinide	7967	5 (0.1%)	5213	1 (0.0%)	5213	1 (0.0%)
DDP4 inhibitors	7967	81 (1.0%)	5213	70 (1.3%)	5213	83 (1.6%)
GLP‐1 receptor agonists	7967	16 (0.2%)	5213	10 (0.2%)	5213	8 (0.2%)
Lipid lowering drugs	7967	4737 (59.5%)	5213	3249 (62.3%)	5213	2689 (51.6%)
Diuretics	7967	2689 (33.8%)	5213	1672 (32.1%)	5213	1388 (26.6%)
Physical examination						
Height (m)	7515	170 (±10.1)	5118	170.5 (±9.8)	5049	170.1 (±9.9)
Weight (kg)						
Mean	7593	87.7 (±17)	5208	86.6 (±15.1)	5203	86.2 (±15.1)
Median		86 (76–97)		85 (75–96)		85 (75–96)
BMI (kg/m^2^)						
Mean	7431	30.3 (±5.3)	5213	29.7 (±4.4)	5213	29.7 (±4.4)
Median		29.8 (26.7–33.2)		29.4 (26.6–32.4)		29.4 (26.6–32.4)
SBP (mmHg)	7813	139.2 (±17.7)	5178	138.5 (±17.0)	5168	139.1 (±17.2)
DBP (mmHg)	7775	77.3 (±10.3)	5151	77.4 (±10.2)	5133	78.4 (±9.6)
Laboratory						
HbA1c (mmol/mol)						
Mean	7716	58.5 (±11.2)	5109	58.4 (±11.2)	5037	48.9 (±8.5)
Median		57 (51–64)		57 (51–64)		48 (43–53)
Total cholesterol (mmol/L)	7553	4.4 (±1.1)	5015	4.4 (±1.0)	4992	4.5 (±1.0)
HDL‐cholesterol (mmol/L)	7529	1.2 (±0.4)	5001	1.2 (±0.3)	4976	1.3 (±0.4)
Cholesterol/ HDL ratio (mmol/L)	7542	3.9 (±1.3)	5011	3.9 (±1.3)	4984	3.7 (±1.2)
LDL‐cholesterol (mmol/L)	7345	2.4 (±0.9)	4889	2.4 (±0.9)	4879	2.5 (±0.9)
Triglycerides (mmol/L)	7452	1.6 (1.2–2.3)	4958	1.6 (1.2–2.3)	4931	1.5 (1.1–2.1)
Serum creatinine (umol/L)	7581	84 (±28.5)	5013	82.5 (±28.0)	4963	79.8 (±24.9)
Lifestyle						
Physical activity (adequate)	5514	2172 (39.4%)	3725	1584 (42.5%)	3729	1803 (48.4%)
Smoking (yes)	7820	1731 (22.1%)	5144	1049 (20.4%)	5120	997 (19.5%)
Alcohol (yes)	4212	729 (17.3%)	2267	404 (17.8%)	2246	481 (21.4%)

At T0, 30.4% used insulin only and 69.6% used a combination of insulin with OGLDs. Of all patients, *n *= 7022 (88.1%) used one type of insulin formulation, that is *n *= 296 (3.7%) used short‐acting insulin, *n *= 2734 (34.3%) used premix insulin, *n *= 2584 (32.4%) used basal insulin, *n *= 1408 (17.7%) used NPH insulin, and *n *= 945 (11.9%) used a combination of insulin types. Mean HbA1c was 58.5 (±11.2) mmol/mol. Mean BMI was 30.3 (±5.3) kg/m^2,^ and 48.6% of patients had a BMI of 30 kg/m^2^ or higher, indicating that almost half of the study group was obese.

The insulin subgroup and matched reference group were comparable regarding sex, age, diabetes duration and BMI. These groups were different regarding HbA1c, as HbA1c is a main driver of the decision to start insulin therapy. Mean HbA1c was 58.4 (±11.2) mmol/mol for the insulin subgroup and 48.9 (±8.5) mmol/mol for the matched references, resulting in a mean difference of 9.5 mmol/mol.

### 3.2 Analysis sets

Weight change data in the first year after index were available for *n *= 5086 patients (ITT analysis set), of which *n *= 4291 patients also used insulin at T+1 (PP analysis set). *N *= 459 patients changed insulin regimen. *N *= 795 patients did not use insulin at T+1 (U < 1y analysis set). Patients included and not included in the ITT analysis set did not differ materially (Table S2).

Availability of weight change data differed for the matched groups. These were available for *n *= 3433 and *n *= 3511 patients of the insulin subgroup (ITT‐insulin set) and matched references (ITT‐reference set), respectively. Weight change data were available for *n *= 2812 matched pairs, referred to as the ITT‐matched analysis set. Of these, the insulin subgroup of *n *= 2349 matched pairs still used insulin at T+1, referred to as the PP‐matched analysis set.

### 3.3 Longitudinal courses

**FIGURE 1:**
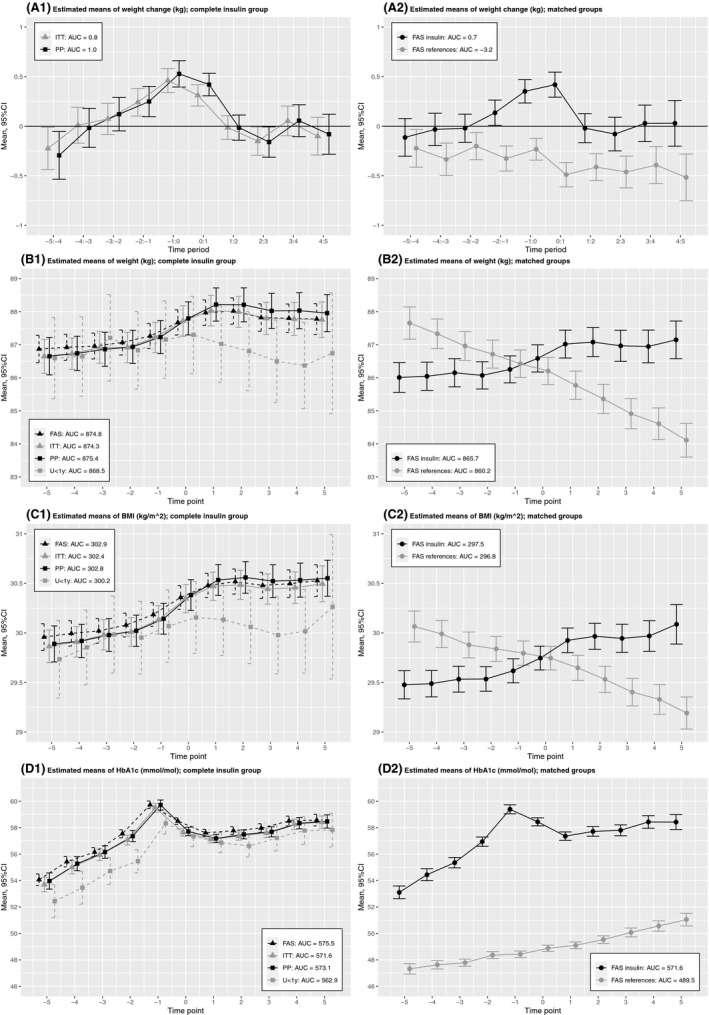
Estimated means with 95% CIs of weight change, weight, BMI and HbA1c in time period T‐5 to T+5, for the complete insulin group and the matched groups. AUC, Area under the curve; FAS, Full analysis set; ITT, Intention‐to‐treat; PP, Per‐protocol; U < 1y, Insulin use less than 1 year

Figure 1 shows estimated means with 95%CIs of weight change, weight, BMI and HbA1c in time period T‐5 to T+5, for both the complete insulin group and the matched groups.

Concerning the ITT analysis set, estimated mean weight changes in the years further than three years prior to index were −0.23 kg (T‐5 to T‐4) and 0.01 kg (T‐4 to T‐3), indicating mean weight loss and no/ minor weight gain, respectively. In the three years prior to index, weight change increased slightly from 0.07 kg (T‐3 to T‐2) and 0.24 kg (T‐2 to T‐1) to 0.46 kg (T‐1 to T0), indicating mean weight gain. Mean weight gain was also present in the first year after index, but at a lower rate with a weight change of 0.31 kg. This pattern is also reflected by the courses of weight and BMI. There were minor differences between the FAS, ITT and PP analysis sets.

In contrast to the insulin subgroup, estimated mean weight changes in the matched reference group were negative in all years concerning the study period, which indicates consistent mean weight loss.

### 3.4 Weight change in the first year

Table 2 shows weight change in the first year after index, including stratified analyses, for both the complete insulin group and matched groups.

**TABLE 2:** Weight change in the time period T0 to T+1 (kg), concerning the complete insulin group and matched groups

		Complete insulin group		Insulin subgroup		Matched references	
		*n*	Mean (±SD) [95%CI]	*n*	Mean (±SD) [95%CI]	*n*	Mean (±SD) [95%CI]
*ITT and ITT‐matched*		5086	0.31 (±3.9) [0.20–0.42]	2812	0.35 (±3.8) [0.21–0.49]	2812	−0.53 (±3.7) [−0.67 to −0.39]
Insulin regimen at T0	Short‐acting	160	0.76 (±4.2) [0.10–1.42]	100	0.99 (±3.5) [0.30–1.68]	100	−0.50 (±4.2) [−1.33–0.34]
	Premixed	1828	0.39 (±3.9) [0.21–0.57]	849	0.51 (±3.9) [0.25–0.77]	849	−0.66 (±3.9) [−0.92 to −0.39]
	Basal	1633	0.23 (±4.0) [0.03–0.42]	1057	0.15 (±4.0) [−0.09–0.39]	1057	−0.67 (±3.6) [−0.89 to −0.45]
	NPH	941	0.28 (±4.6) [0.05–0.51]	555	0.37 (±3.4) [0.08–0.66]	555	−0.15 (±3.6) [−0.45–0.15]
	Combinations	524	0.24 (±4.3) [−0.13–0.60]	251	0.39 (±3.8) [−0.07–0.86]	251	−0.40 (±3.5) [−0.83–0.04]
BMI at T0	<25	639	0.97 (±3.2) [0.71–1.22]	332	1.02 (±2.9) [0.70–1.33]	338	0.08 (±2.9) [−0.22–0.39]
	≥25–<30	1914	0.50 (±3.4) [0.34–0.65] ^L^*	1206	0.46 (±3.5) [0.26–0.66]	1206	−0.54 (±3.4) [−0.73 to −0.36] ^L^*
	≥30	2425	0.03 (±4.4) [−0.15–0.20] ^L^***^, M^***	1274	0.08 (±4.3) [−0.16–0.31] ^L***, M*^	1268	−0.68 (±4.2) [−0.92 to −0.45] ^L^**
HbA1c at T0	<53	1620	0.23 (±4.0) [0.04–0.42]	906	−0.31 (±3.7) [0.07–0.55]	2060	−0.39 (±3.8) [−0.55 to −0.22]
	≥53–<61	1709	0.13 (±3.7) [−0.05–0.31]	973	0.23 (±3.7) [0.00–0.46]	497	−0.99 (±3.3) [−1.29 to −0.70] ^L^**
	≥61	1639	0.56 (±4.1) [0.36–0.75] ^M^**	892	0.56 (±4.1) [0.29–0.83]	165	−1.28 (±4.1) [−1.91 to −0.65] ^L^**
*PP and PP‐matched*		4291	0.42 (±3.8) [0.31–0.53]	2349	0.47 (±3.7) [0.32–0.62]	2349	−0.51 (±3.7) [−0.66 to −0.36]
Insulin regimen at T0	Short‐acting	115	0.70 (±4.5) [−0.14–1.53]	75	0.93 (±3.7) [0.09–1.77]	75	−0.44 (±4.2) [−1.40–0.52]
	Premixed	1579	0.50 (±3.7) [0.32–0.69]	734	0.63 (±3.7) [0.36–0.91]	734	−0.70 (±3.9) [−0.98 to −0.41]
	Basal	1368	0.37 (±3.8) [0.17–0.58]	879	0.28 (±3.9) [0.02–0.54]	879	−0.63 (±3.6) [−0.87 to −0.39]
	NPH	772	0.42 (±3.4) [0.18–0.66]	442	0.51 (±3.3) [0.20–0.82]	442	0.02 (±3.5) [−0.30–0.35]
	Combinations	457	0.20 (±4.2) [−0.19–0.59]	219	0.43 (±3.7) [−0.06–0.92]	219	−0.47 (±3.5) [−0.93 to −0.02]
BMI at T0	<25	519	1.01 (±3.1) [0.74–1.28]	274	1.07 (±3.0) [0.71–1.42]	277	0.10 (±3.0) [−0.25–0.46]
	≥25–<30	1634	0.57 (±3.3) [0.41–0.73]	1015	0.56 (±3.4) [0.36–0.77]	1016	−0.54 (±3.4) [−0.75 to −0.33] ^L^*
	≥30	2048	0.17 (±4.3) [−0.01–0.36] ^L^***^, M^**	1060	0.23 (±4.2) [−0.03–0.48] ^L^**	1056	−0.64 (±4.2) [−0.89 to −0.39] ^L^**
HbA1c at T0	<53	1335	0.28 (±3.8) [0.07–0.48]	743	0.32 (±3.6) [0.06–0.58]	1725	−0.38 (±3.8) [−0.56 to −0.20]
	≥53–<61	1473	0.27 (±3.6) [0.08–0.45]	823	0.39 (±3.5) [0.15–0.63]	411	−1.00 (±3.4) [−1.32 to −0.67] ^L^**
	≥61	1375	0.70 (±3.9) [0.49–0.90] ^L^*^, M^**	746	0.75 (±4.0) [0.47–1.04]	131	−1.09 (±4.1) [−1.79 to −0.39]

Note

One‐way ANOVA and post‐hoc analyses, using Bonferroni‐adjusted p‐values, were performed: In the complete insulin group, there was no significant difference between insulin category, whereas there was between BMI category (p < .001 for ITT and PP) and HbA1c tertile (p = .005 for ITT and p = .003 for PP).

In the insulin subgroup, there was no significant difference between insulin category, whereas there was between BMI category (p < .001 for ITT‐matched and p = .02 for PP‐matched) and HbA1c tertile (p = .005 for ITT‐matched and p = .003 for PP‐matched).

In the matched references, there was a significant difference between BMI category (p = .004 for ITT‐matched and p = .012 for PP‐matched) and HbA1c tertile (p < .001 for ITT matched and p = .002 for PP‐matched).

Results of post hoc tests are given in the table: ^L^, Compared to the lowest category; ^M^, Compared to the middle category; *, p‐adjusted < 0.05; **, p‐adjusted < 0.01; ***, p‐adjusted < 0.001.

Concerning the ITT analysis set, mean weight gain was 0.31 (±3.9) kg [95%CI: 0.20 kg–0.42 kg]. When categorized, *n *= 2425 patients gained weight, which equals an absolute risk for weight gain of 47.7% [95%CI: 46.3%–49.1%] (Figure S2). Moreover, *n *= 509 patients (10.0%) gained a substantial 5 kg weight or more. Mean weight gain in the PP analysis set was slightly higher, notably 0.42 (±3.8) kg [95%CI: 0.31 kg–0.53 kg].

There was no statistically significant difference in weight change between insulin regimens, whereas there was between HbA1c tertile and BMI category. No linear association was found, but patients in the highest HbA1c tertile gained the most weight. BMI category was inversely associated with weight change.

Table S3a presents results of the sensitivity analysis concerning weight change in the first year prior to index (T‐1 to T0) showing 0.43 kg. This indicates that mean weight gain in the first year after actual initiation of insulin therapy would probably be somewhere in between 0.31 kg and 0.43 kg.

In contrast to the insulin subgroup, matched references on average lost weight. Importantly, in matched references, HbA1c was inversely associated with weight change. Similar to the insulin subgroup, an inverse association of BMI with weight change was found.

### 3.5 Effect of insulin therapy on body weight

Based on univariate analysis, weight change T‐2 to T0 in kg was chosen as main covariate (Table S4, Figure S3). In both the insulin subgroup and matched references, weight change T‐2 to T0 was inversely associated with weight change in the first year after index. Explained variance was approximately 5%, the highest of all studied variables. Additional covariates were selected based on Table S5 and Table S6.

Univariate and multivariable regression analysis, on the comparison of matched groups, is shown in Table 3. Concerning the ITT‐matched analysis sets, the insulin subgroup was associated with weight increase after index (β = 0.884, p < .001). When adjusted for pre‐insulin weight change T‐2 to T0, insulin treatment initiation remained associated with weight increase (β‐adjusted = 1.288, p < .001). This indicates that mean weight change was 1.3 kg higher in patients that initiated insulin therapy compared to their matched references. The association was the strongest in the PP‐matched analysis set (β‐adjusted = 1.448, p < .001). After adjusting for additional covariates, both pre‐insulin weight change T‐2 to T0 and insulin treatment initiation retained statistical significance (p < .001).

**TABLE 3:** Linear regression analysis on weight change (kg) in time period T0 to T+1, concerning comparison of the matched groups (ITT‐matched: *n *= 2812 pairs and PP‐matched: *n *= 2349 pairs)

	ITT‐matched analysis set				PP‐matched analysis set			
	*n*	Beta (SE)	p‐value	*R* ^2^	*n*	Beta (SE)	p‐value	*R* ^2^
Model 1. Univariate								
Group (insulin subgroup)	5624	0.884 (0.101)	<.001	0.013	4698	0.977 (0.108)	<.001	0.017
Model 2. Model 1 + T<0								
Group (insulin subgroup)	3594	1.288 (0.121)	<.001	0.072	2976	1.448 (0.132)	<.001	0.075
Weight change T‐2 to T0 (kg)		−0.175 (0.013)	<.001			−0.169 (0.014)	<.001	
Model 3. Model 2 + weight								
Group (insulin subgroup)	3594	1.287 (0.121)	<.001	0.073	2976	1.448 (0.132)	<.001	0.077
Weight change T‐2 to T0 (kg)		−0.171 (0.013)	<.001			−0.164 (0.014)	<.001	
Weight		−0.008 (0.004)	.049			−0.011 (0.005)	.019	
Model 4. Model 3 + T0								
Group (insulin subgroup)	3594	1.175 (0.138)	<.001	0.068	2976	1.468 (0.151)	<.001	0.080
Weight change T‐2 to T0 (kg)		−0.163 (0.015)	<.001			−0.155 (0.016)	<.001	
Weight		−0.013 (0.005)	.009			−0.017 (0.005)	.001	
Metformin						−0.266 (0.155)	.086	
Diuretics		−0.300 (0.147)	.042					
Model 5. Model 4 + T>0								
Group (insulin subgroup)	3507	1.177 (0.176)	<.001	0.103	1745	1.349 (0.194)	<.001	0.106
Weight change T‐2 to T0 (kg)		−0.165 (0.016)	<.001			−0.160 (0.018)	<.001	
Weight		−0.013 (0.005)	.013			−0.018 (0.006)	.002	
HbA1c change T0 to T+1		0.050 (0.011)	<.001			0.046 (0.011)	<.001	
Metformin initiated T0 to T+1		−0.717 (0.326)	.028					
Sulphonylureas stopped T0 to T+1		−0.837 (0.273)	.002			−0.844 (0.316)	.008	
Diuretics stopped T0 to T+1		−0.551 (0.323)	.088					
Activity decrease T0 to T+1						−0.511 (0.264)	.053	
HbA1c T+1		0.023 (0.010)	.022			0.022 (0.011)	.042	

Note

Input variables for model 4 (backward regression) were: Group, pre‐insulin weight change T‐2 to T0, weight, hbA1c, metformin, diuretics, physical activity.

Input variables for model 5 (backward regression) were: Group, pre‐insulin weight change T‐2 to T0, weight, hbA1c, metformin, diuretics, physical activity, hbA1c change T0 to T+1, Metformin stopped T0 to T+1, Metformin initiated T0 to T+1, Sulphonylurea stopped T0 to T+1, Sulphonylurea initiated T0 to T+1, Diuretics stopped T0 to T+1, Diuretics initiated T0 to T+1, Physical activity decrease T0 to T+1, Physical activity increase T0 to T+1, and hbA1c T+1.

### 3.6 Stratified analysis

**FIGURE 2:**
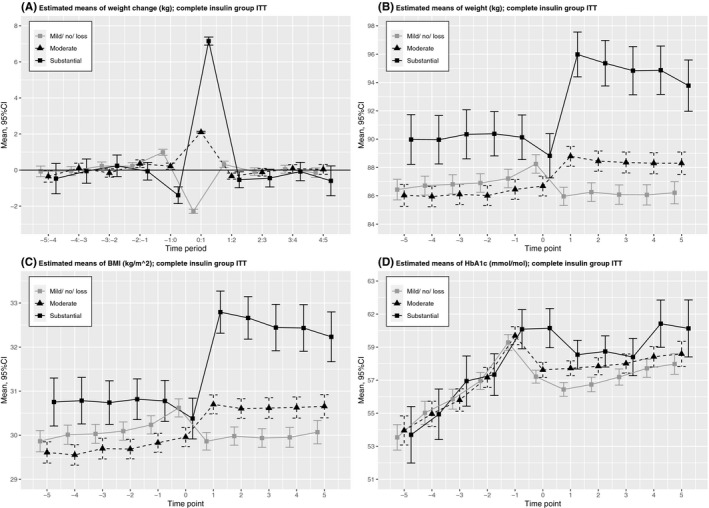
Estimated means with 95%CIs of weight change, weight, BMI and HbA1c in time period T‐5 to T+5, stratified by weight change category

In the first year after index, substantial weight gain was seen in *n *= 509 patients (10.0%), moderate weight gain was seen in *n *= 1916 patients (37.7%), and mild gain/ no change/ weight loss was seen in *n *= 2661 patients (52.3%). Figure 2 shows that patients with substantial weight gain had higher estimated mean BMI values prior to T‐1 as compared to the other categories. Estimated mean weight and BMI decreased in the year prior to index, of which BMI decreased to a value within the range of the other categories at T0. Simultaneously, estimated mean HbA1c remained unaltered high at T0. Correlations between weight change T‐2 to T0 and weight change the first year after index were *r* = −0.172 (p = .002) for the substantial subgroup (*n *= 322), *r* = −0.029 (p = .319) for the moderate subgroup (*n *= 1181) and *r* = −0.204 (p < .001) for the mild gain/ no change/ weight loss subgroup (*n *= 1537).

## 4 DISCUSSION

This study showed that estimated mean weight gradually increased in the years prior to insulin therapy and continued to increase in the first year after, albeit at a slightly lower rate. Pre‐insulin weight change was inversely associated with weight change in the first year after, with the highest statistical explained variance of all modelled covariates. Initiation of insulin therapy was associated with weight increase, independent of pre‐insulin weight change and additional covariates. Moreover, compared to the ITT‐matched analysis (assessment of treatment policy), the associations in the PP‐matched analysis (assessment of the true effect of a drug) were the strongest. Mean weight gain in the first year after insulin therapy, however, was small and subject to substantial variation. Patients with substantial weight gain showed high initial pre‐insulin estimated mean weight/ BMI values, and mean weight/ BMI loss in the year preceding insulin.

Few other studies included pre‐insulin weight change.^9^, ^16^, ^17^ Of these, Gordon et al^9^ visualized a more or less stable weight prior to insulin therapy.^9^ Another observational study by Gant et al,^16^ in not necessarily naïve insulin users referred to secondary care, reported a similar‐shaped curve concerning averaged BMI course.^16^ The averaged BMI course did not significantly differ between insulin users and non‐insulin users (p‐interaction* *= 0.460).

In the present study, we adjusted insulin therapy initiation for pre‐insulin weight change, showing that insulin therapy is independently associated with weight increase in the first year after.

In the study by Larger et al,^17^ all patients lost weight prior to insulin therapy due to unreported cause, followed by initiation of insulin therapy with delineating weight gain.^17^ Weight gain after insulin therapy initiation was correlated with prior maximum lifetime weight and maximal daily insulin dose,^17^ resulting in the hypothesis that weight gain observed after the introduction of insulin may mostly be catch‐up weight gain.^26^

In the present study, by stratification, we showed that only those patients with substantial weight gain had estimated mean weight loss in the year preceding insulin. This subgroup showed an estimated mean BMI decrease, which at T0 reached a value within the range of the other subgroups. Combined with an unaltered high estimated mean HbA1c at T‐1 and T0, these results may indicate poor glucose control with subsequent increased glycosuria resulting in weight loss.^2^

An alternative explanation for pre‐insulin weight loss could be that, by losing weight, patients attempted to postpone insulin therapy. However, pre‐insulin estimated mean weight loss was not found in the other categories. Concerning matched references, attempting to postpone insulin therapy could explain the finding that patients in the highest HbA1c tertile lost the most weight.

Determinants of substantial weight gain after the initiation of insulin therapy will be further investigated in the second stage of this study.

Several studies, including the present one, found an inverse association of baseline weight/ BMI with weight change after initiation.^6^, ^8^, ^10^, ^11^, ^12^, ^13^, ^14^ In contrast with general clinical belief, reassurance with regard to the use of insulin in obese patients was reported.^6^, ^11^ In this study, we showed that patients with substantial weight gain had a more or less similar estimated mean BMI at baseline, but markedly higher estimated BMI values prior to baseline (T‐5 to T‐1). This novel finding indicates that using baseline BMI as a determinant of weight gain after insulin therapy initiation may be misleading.

### 4.1 Mean weight gain after insulin initiation

A mean weight gain of 0.31 kg to 0.43 kg in the first year after initiation of insulin therapy is small as compared to several others reports. Of presented studies,^5^, ^6^, ^7^, ^8^, ^9^, ^10^, ^11^, ^12^, ^13^, ^14^ mean one‐year weight gains of 0.98 ± 7.1 kg,^7^ 1.2 kg,^10^ 1.78 to 2.00 kg,^5^, ^6^ 2.8 ± 6.0 kg^9^ and 3 kg^13^ were reported.

Differences in weight gain may be attributable to various, whether or not coherent, factors, for example patient characteristics, study medication including dose, co‐medication, analysis sets and study setting. The CREDIT study, a multinational study, showed that mean weight gain substantially differs between countries, ranging from 0.95 kg in Germany to 4.26 kg in Portugal.^6^ Also, regional differences within the United Kingdom were reported.^12^

### 4.2 The Netherlands

In the Netherlands, everyone has a general practitioner and over 80% of patients with T2DM is treated in primary care. Patients were treated according to the Dutch College of General Practitioner Guideline of the study period, advising to start insulin therapy when HbA1c is at 53 mmol/mol (7%), allowing adjustment with higher cutoff points in the elderly and frail.^19^ Moreover, it was advised that lifestyle and diet aspects needed to be considered with each treatment step intensification. NPH insulin (8–12 units Insulatard), in addition to OGLDs, is the first choice of treatment. Table 1, however, shows that the majority of patients were prescribed long‐acting insulin analogue, which is a phenomenon previously reported by Barnett et al^22^ Furthermore, the relatively high percentage of insulin‐only users (30.4%) is an unexpected finding.

The majority of contacts and treatment decisions are through or by practice nurses, specifically trained to provide care to patients with T2DM. This training emphasizes application of strict adherence to countrywide accepted protocols and treatments steps. This approach minimizes delays in the decision time to next treatment steps, thus counteracting clinical inertia.

### 4.3 Generalizability

In general, the results found in ZODIAC are considered representative for the Netherlands, and therefore generalizable, at least for that part of the Dutch population with a Caucasian background. Due to the geographical distribution of the primary care practices participating in ZODIAC, patients of Moroccon, Turkish, Hindoustani or Surinam descent are decidedly under‐represented.

### 4.4 Strengths and limitations

Strengths and limitations inherent to the study design were previously described.^6^, ^20^, ^23^ The effect of insulin was studied in routine clinical practice, which allows for real‐life information on use in the general population. As data were gathered annually, the exact time of insulin therapy initiation could not be identified. Also, we cannot exclude the possibility of some registration delay, which we believe has a maximum of one year. Furthermore, we cannot exclude the possibility that some patients in the insulin‐only group were also using OGLDs; chances are that this will only be the case in a minority of patients. The clinical characteristics of the insulin‐only users were barely different from the patients treated with a combination of insulin and OGLDs (data not shown).

Insulin doses, frequency of injections and nutritional factors were not included in ZODIAC. Also, study site was not taken into account.

Weight was measured to the nearest kg, hence more as a discrete than a continuous variable. Body fat distribution was not measured.^27^ Furthermore, weight change cannot be attributed to fat tissue only, as an unmeasured fat free component notably fluid may play a role in weight change.^24^ Moreover, no patient selections other than those described in the methods section were made; hence, the group also included some patients with clinically relevant renal disease (a rare patient group in the primary care in the Netherlands) and patients with chronic heart failure. Since no structural information on fluid status or degree of chronic heart failure was available, the influence of these factors could not be taken into account.

### 4.5 Conclusion

We conclude that initiation of insulin therapy was independently associated with weight increase; however, overall effect on weight was small and subject to substantial variation. Pre‐insulin weight change is identified as a relatively strong inverse determinant of weight change after insulin initiation.

## CONFLICT OF INTEREST

The authors have nothing to declare.

## AUTHOR CONTRIBUTIONS

MAE obtained the idea for the study, designed the study, researched data and wrote the manuscript, PRvD contributed to the discussion and reviewed/edited the manuscript, EH co‐designed the study, contributed to the discussion and reviewed/edited the manuscript, and HJGB obtained the idea for the study, designed the study, contributed to the discussion and reviewed/edited the manuscript.

## Supporting information

Supplementary Material

## Data Availability

Data available on request from the authors.

## References

[1] Inzucchi SE , Bergenstal RM , Buse JB , et al. Management of hyperglycaemia in type 2 diabetes, 2015: A patient‐centred approach. update to a position statement of the american diabetes association and the european association for the study of diabetes. Diabetologia. 2015;58(3):429‐442. 10.1007/s00125-014-3460-0 25583541

[2] Brown A , Guess N , Dornhorst A , Taheri S , Frost G . Insulin‐associated weight gain in obese type 2 diabetes mellitus patients: What can be done? Diabetes Obes Metab. 2017;19(12):1655‐1668. 10.1111/dom.13009 28509408

[3] Dimitriadis G , Mitrou P , Lambadiari V , Maratou E , Raptis SA . Insulin effects in muscle and adipose tissue. Diabetes Res Clin Pract. 2011;93(Suppl 1):52. 10.1016/S0168-8227(11)70014-6 21864752

[4] Erpeldinger S , Rehman MB , Berkhout C , et al. Efficacy and safety of insulin in type 2 diabetes: Meta‐analysis of randomised controlled trials. BMC Endocrine Disorders. 2016;16(1):39‐z. 10.1186/s12902-016-0120-z 27391319 PMC4939045

[5] Balkau B , Calvi‐Gries F , Freemantle N , Vincent M , Pilorget V , Home PD . Predictors of HbA1c over 4 years in people with type 2 diabetes starting insulin therapies: The CREDIT study. Diabetes Res Clin Pract. 2015;108(3):432‐440. 10.1016/j.diabres.2015.02.034 25818885

[6] Balkau B , Home PD , Vincent M , Marre M , Freemantle N . Factors associated with weight gain in people with type 2 diabetes starting on insulin. Diabetes Care. 2014;37(8):2108‐2113. 10.2337/dc13-3010 24824546

[7] Bramlage P , Bluhmki T , Fleischmann H , et al. Determinants of weight change in patients on basal insulin treatment: An analysis of the DIVE registry. BMJ Open Diabetes Research & Care. 2017;5(1):e000301. 10.1136/bmjdrc-2016-000301 PMC5278215 28176957

[8] Dornhorst A , Luddeke HJ , Sreenan S , et al. Insulin detemir improves glycaemic control without weight gain in insulin‐naive patients with type 2 diabetes: Subgroup analysis from the PREDICTIVE study. Int J Clin Pract. 2008;62(4):659‐665. 10.1111/j.1742-1241.2008.01715.x 18324957

[9] Gordon J , Pockett RD , Tetlow AP , McEwan P , Home PD . A comparison of intermediate and long‐acting insulins in people with type 2 diabetes starting insulin: An observational database study. Int J Clin Pract. 2010;64(12):1609‐1618. 10.1111/j.1742-1241.2010.02520.x 20946269 PMC3036815

[10] Jansen HJ , Hendriks JC , de Galan BE , Penders G , Tack CJ , Vervoort G . Contribution of change in glycosylated haemoglobin to insulin‐associated weight gain: Results of a longitudinal study in type 2 diabetic patients. Endocrine. 2011;39(2):190‐197. 10.1007/s12020-010-9423-4 21069577

[11] Paul SK , Shaw JE , Montvida O , Klein K . Weight gain in insulin‐treated patients by body mass index category at treatment initiation: New evidence from real‐world data in patients with type 2 diabetes. Diabetes Obes Metab. 2016;18(12):1244‐1252. 10.1111/dom.12761 27502528

[12] Watson L , Wilson BP , Alsop J , Kumar S . Weight and glycaemic control in type 2 diabetes: What is the outcome of insulin initiation? Diabetes Obes Metab. 2011;13(9):823‐831. 10.1111/j.1463-1326.2011.01413.x 21481128

[13] Yadgar‐Yalda R , Colman PG , Fourlanos S , Wentworth JM . Factors associated with insulin‐induced weight gain in an australian type 2 diabetes outpatient clinic. Intern Med J. 2016;46(7):834‐839. 10.1111/imj.13122 27130246

[14] Zekarias K , Davey C , Seaquist E . Intensification of medical management in type 2 diabetes: A real‐world look at primary care practice. J Diabetes Complications. 2020;34(1):107477. 31711841 10.1016/j.jdiacomp.2019.107477 PMC6920559

[15] Cichosz SL , Lundby‐Christensen L , Johansen MD , et al. Prediction of excessive weight gain in insulin treated patients with type 2 diabetes. J Diabetes. 2017;9(4):325‐331. 10.1111/1753-0407.12418 27130075

[16] Gant CM , Mensink I , Binnenmars SH , et al. Body weight course in the DIAbetes and LifEstyle cohort twente (DIALECT‐1)‐A 20‐year observational study. PLoS One. 2019;14(6):e0218400. 10.1371/journal.pone.0218400 31216324 PMC6583961

[17] Larger E , Rufat P , Dubois‐Laforgue D , Ledoux S . Insulin therapy does not itself induce weight gain in patients with type 2 diabetes. Diabetes Care. 2001;24(10):1849‐1850. 10.2337/diacare.24.10.1849 11574460

[18] Ubink‐Veltmaat LJ , Bilo HJ , Groenier KH , Houweling ST , Rischen RO , Meyboom‐de Jong B . Prevalence, incidence and mortality of type 2 diabetes mellitus revisited: A prospective population‐based study in the netherlands (ZODIAC‐1). Eur J Epidemiol. 2003;18(8):793‐800. 12974556 10.1023/a:1025369623365

[19] Rutten G , De Grauw W , Nijpels G , et al. NHG‐standaard diabetes mellitus type 2 (tweede herziening). Huisarts Wet. 2006;49(3):137:52.

[20] Ray WA . Evaluating medication effects outside of clinical trials: New‐user designs. Am J Epidemiol. 2003;158(9):915‐920. 10.1093/aje/kwg231 14585769

[21] Sacks DB . Measurement of hemoglobin A(1c): A new twist on the path to harmony. Diabetes Care. 2012;35(12):2674‐2680. 10.2337/dc12-1348 23173136 PMC3507560

[22] Barnett A , Begg A , Dyson P , Feher M , Hamilton S , Munro N . Insulin for type 2 diabetes: Choosing a second‐line insulin regimen. Int J Clin Pract. 2008;62(11):1647‐1653. 10.1111/j.1742-1241.2008.01909.x 19143853 PMC2680733

[23] Looker HC , Knowler WC , Hanson RL . Changes in BMI and weight before and after the development of type 2 diabetes. Diabetes Care. 2001;24(11):1917‐1922. 11679457 10.2337/diacare.24.11.1917

[24] Packianathan IC , Fuller NJ , Peterson DB , Wright A , Coward WA , Finer N . Use of a reference four‐component model to define the effects of insulin treatment on body composition in type 2 diabetes: The 'darwin study'. Diabetologia. 2005;48(2):222‐229. 10.1007/s00125-004-1642-x 15688205

[25] Hartman YAW , Jansen HJ , Hopman MTE , Tack CJ , Thijssen DHJ . Insulin‐associated weight gain in type 2 diabetes is associated with increases in sedentary behavior. Diabetes Care. 2017;40(9):e120‐e121. 10.2337/dc17-0787 28694305

[26] Larger E . Weight gain and insulin treatment. Diabet Metab 2005;31(4):4S51–4S56. 10.1016/S1262-3636(05)88268-0 16389899

[27] Lee JJ , Pedley A , Hoffmann U , Massaro JM , Levy D , Long MT . Visceral and intrahepatic fat are associated with cardiometabolic risk factors above other ectopic fat depots: The framingham heart study. Am J Med. 2018;131(6):684‐692.e12. 10.1016/j.amjmed.2018.02.002 29518370 PMC5964004

