# Supplementary material for: Course of body weight before and after the initiation of insulin therapy in type 2 diabetes mellitus: Retrospective inception cohort study (ZODIAC 58)
Source: Endocrinol Diabetes Metab. 2020 Dec 7;4(2):e00212. doi: 10.1002/edm2.212 (PMC8029529; doi:10.1002/edm2.212)
Supplement: Supplementary file 1 — Supplementary Material [file EDM2-4-e00212-s001.docx]

**Supplementary Appendix**

Course of body weight before and after the initiation of insulin therapy in type 2 diabetes mellitus: retrospective inception cohort study (ZODIAC 58)

**CONTENTS (9)**

**Figure S1.** Study design; Patient selection and subsequent data collection. 2

**Table S1.** Data cleaning 3

**Table S2.** Comparison of patient included and not included in the ITT analysis set, 4

concerning the complete insulin group

**Figure S2.** Distribution of weight change in time period T0 to T+1 (kg and %), 5

concerning the ITT analysis set of the complete insulin group (n=5086).

**Table S3.** Weight change in time period T-1 to T0 (kg) 6

- 3a. Complete insulin group
- 3b. Matched groups

**Table S4.** Explorative Pearson correlation analysis with weight change (kg) T0 – T+1 7

**Figure S3.** Association of weight change in time period T-2 to T0 (x-axis) with weight change 8

T0 to T+1 in kg and % (y-axis), for both the complete insulin group and the matched groups

**Table S5.** Comparison of patients, concerning the ITT-matched analysis set 9

**Table S6.** Univariate regression analysis concerning the ITT-matched analysis set 10

**
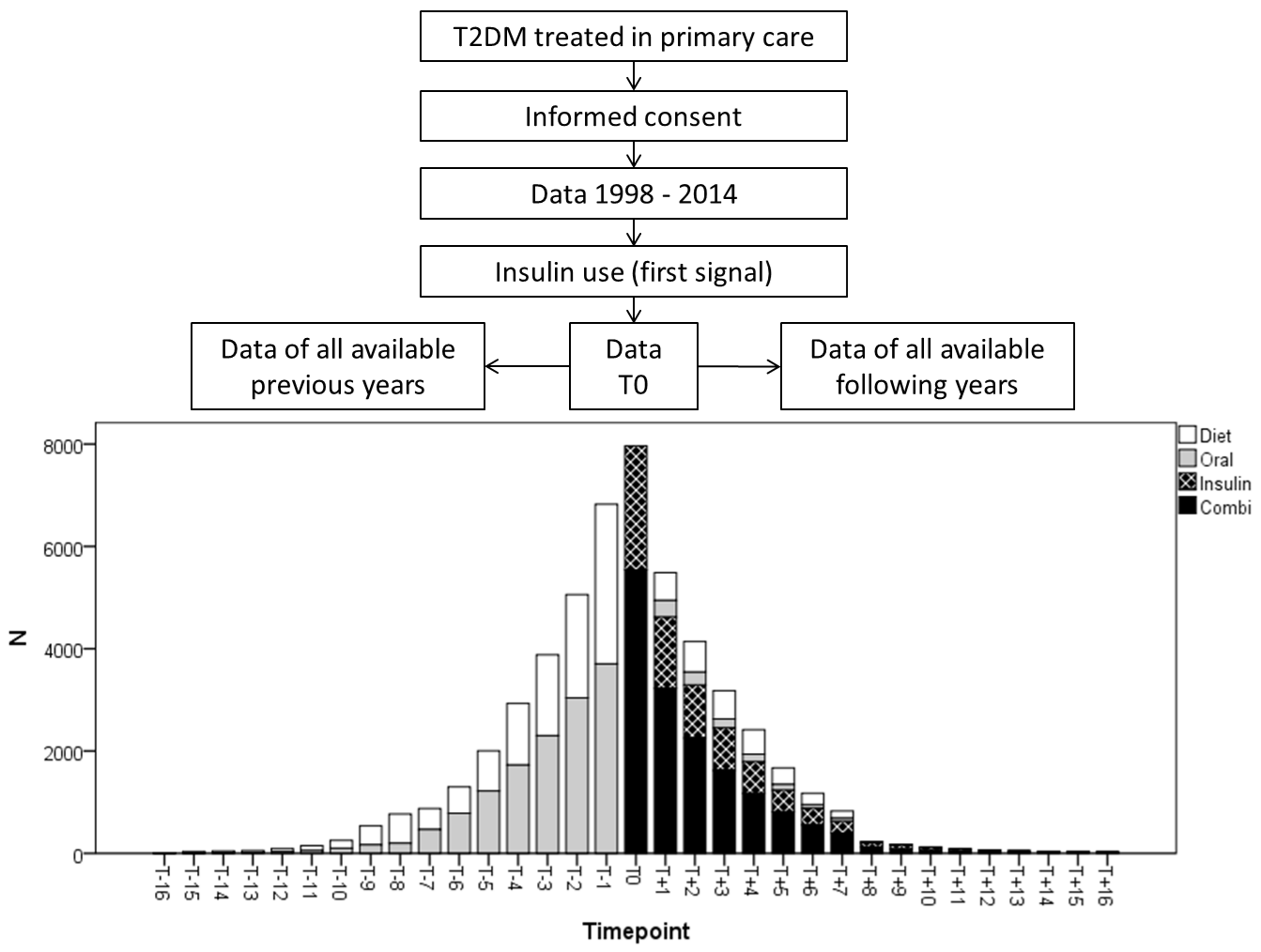
**

**Figure S1.** Study design; Patient selection and subsequent data collection.

The embedded bar chart shows the number of patients present at each time point, including the distribution of the four main treatment groups: “diet”, “OGLDs”, “insulin”, and “combination of OGLDs and insulin”.

Data analysis was limited to T-5 to T+5.

Combi, combination of oral glucose lowering drugs and insulin.

**Table S1.** Data cleaning.

| ***Clinically unlikely data points were excluded from analysis:*** |
| --- |
| HbA1c concentrations exceeding 130 mmol/mol, BMI outside the range 16 – 73 kg/m² |
| Height outside the range 50 – 220 cm |
| Weight outside the range 30 – 200 kg |
| Weight change per year outside the range -20% – +20% (1) |
| Age at diagnosis earlier than 20 years, and subsequent diabetes duration |
| As LDL cholesterol was calculated by Friedewald’s formula (2), we excluded LDL cholesterol when triglycerides exceeded 8 mmol/ l |

**Table S2.** Comparison of patient included and not included in the ITT analysis set,

concerning the complete insulin group (sensitivity analysis).

|  | Included in ITT  (n=5085) | | Not included in ITT  (n=2881) | |
| --- | --- | --- | --- | --- |
|  | n | Summary statistics | n | Summary statistics |
| Age (years) | 5085 | 68.2 (±11.2) | 2881 | 69.8 (±12.5) |
| Sex (man) | 5085 | 2387 (46.9%) | 2881 | 1343 (46.6%) |
| Age at diagnosis (years) | 5013 | 58.2 (±11.1) | 2829 | 59.3 (±12.5) |
| Diabetes duration (years) | 5013 | 9 (6 – 13) | 2829 | 9 (6 – 14) |
| BMI | 4978 | 30.3 (±5.2) | 2453 | 30.3 (±5.6) |
| HbA1c | 4968 | 57.7 (±10.6) | 2748 | 60.0 (±12.1) |
| Main category   - OGLDs and insulin - Insulin only | 5085 | 3628 (71.3%)  1458 (28.7%) | 2881 | 1914 (66.4%)  967 (33.6%) |
| Metformin | 5086 | 3179 (62.5%) | 2881 | 1659 (57.6%) |
| Lipid lowering drugs | 5086 | 3146 (61.9%) | 2881 | 1591 (55.2%) |
| Diuretics | 5086 | 1705 (33.5%) | 2881 | 984 (34.2%) |
| Insulin regimen   - Short-acting - Premixed - Basal - NPH - Combinations | 5085 | 160 (3.1%)  1828 (35.9%)  1633 (32.1%)  941 (18.5%)  524 (10.3%) | 2881 | 136 (4.7%)  906 (31.4%)  951 (33.0%)  467 (16.2%)  421 (14.6%) |

**Figure S2.** Distribution of weight change in time period T0 to T+1 (kg and %), concerning the ITT analysis set of the complete insulin group (n=5086).

| 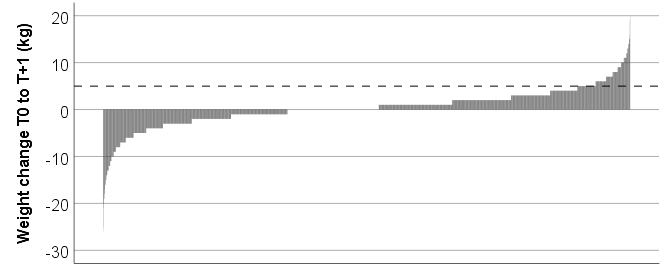  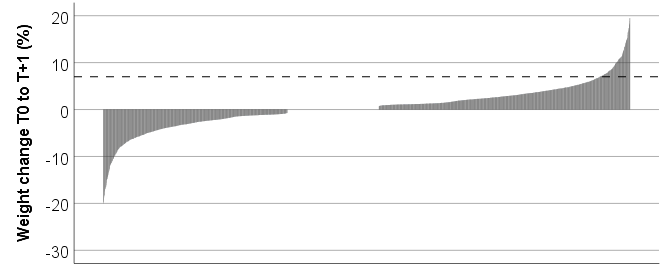 |
| --- |

**Interpretation:** A weight change value above zero indicates weight increase, and vice versa a weight change value below zero indicates weight decrease.

When categorized, n=1777 patients (34.9%) lost weight, n=884 patient (17.4%) remained stable weight, and n=2425 patients (47.7%) gained weight. Mean weight gain was 0.31 ±3.9 kg.

Reference lines at 5 kg (3) and at 7% (4) were included, for comparison with the literature. Of the ITT analysis set, n=509 patients (10.0%, 95%CI: 9.2% − 10.9%) gained 5 kg weight or more and n=291 patients (5.7%, 95%CI: 5.1% - 6.4%) gained more than 7% weight.

**Table S3a.** Weight change (kg) in time period T-1 to T0, for the complete insulin group

(sensitivity analysis).

|  | n | Mean (sd) [95% CI] |
| --- | --- | --- |
| FAS analysis set | 6159 | 0.38 (±4.1) [0.28 – 0.49] |
| ITT analysis set | 4211 | 0.43 (±4.0) [0.31 – 0.56] |
| PP analysis set | 3548 | 0.50 (±4.1) [0.37 – 0.63] |

**Table S3b.** Weight change (kg) in time period T-1 to T0, for the matched groups

(sensitivity analysis).

|  | **Insulin subgroup** | | **Matched references** | |
| --- | --- | --- | --- | --- |
|  | n | Mean (±sd) [95% CI] | n | Mean (±sd) [95% CI] |
| FAS analysis sets | 4435 | 0.34 (±4.0) [0.23 – 0.46] | 4058 | -0.23 (±3.9) [-0.34 – -0.12] |
| lTT analysis sets | 2972 | 0.40 (±3.9) [0.26 – 0.54] | 2828 | -0.20 (±3.4) [-0.33 – -0.07] |
| ITT-matched analysis set | 2435 | 0.39 (±4.0) [0.23 – 0.55] | 2277 | -0.15 (±3.5) [-0.30 – -0.01] |
| PP-matched analysis set | 2021 | 0.43 (±4.1) [0.25 – 0.61] | 1892 | -0.15 (±3.5) [-0.31 – 0.01] |

**Table S4.** Explorative Pearson correlation analysis with weight change (kg) in time period T0 – T+1.

|  | **Complete insulin group (ITT)** | | | **Insulin subgroup (ITT)** | | | **Matched references (ITT)** | | | |
| --- | --- | --- | --- | --- | --- | --- | --- | --- | --- | --- |
|  | n | r | p-value | n | r | p-value | n | r | | p-value |
| **Time point T0** |  |  |  |  |  |  |  |  | |  |
| Weight (kg) | 5086 | **-**0.096 | <0.001 | 3433 | **-**0.088 | <0.001 | 3511 | **-**0.046 | | 0.006 |
| BMI (kg/m^2^) | 4978 | **-**0.118 | <0.001 | 3433 | **-**0.099 | <0.001 | 3511 | **-**0.053 | | 0.002 |
| HbA1c (mmol/mol) | 4968 | 0.058 | <0.001 | 3367 | 0.063 | <0.001 | 3393 | **-**0.113 | | <0.001 |
| **Time period T-1 to T0** |  |  |  |  |  |  |  |  | |  |
| Weight change (kg) | 4211 | **-**0.208 | <0.001 * | 2972 | **-**0.215 | <0.001 * | 2828 | **-**0.175 | | <0.001 * |
| BMI change (kg/m^2^) | 4038 | **-**0.165 | <0.001 * | 2899 | **-**0.167 | <0.001 * | 2754 | **-**0.147 | | <0.001 * |
| HbA1c change (mmol/mol) | 4201 | 0.045 | 0.004 * | 2974 | 0.045 | 0.014 * | 2765 | **-**0.086 | | <0.001 * |
| **Time period T-2 to T0** |  |  |  |  |  |  |  |  | |  |
| HbA1c change (mmol/mol) | 2973 | 0.034 | 0.063 ^#^ | 2159 | 0.058 | 0.007 * | 2157 | **-**0.090 | | <0.001 * |
| Averaged HbA1c change (mmol/mol) | 2593 | 0.035 | 0.072 ^#^ | 1921 | 0.061 | 0.008 * | 2029 | **-**0.097 | | <0.001 * |
| BMI change (kg/m^2^) | 2900 | **-**0.195 | <0.001 * | 2122 | **-**0.190 | <0.001 * | 2142 | **-**0.181 | | <0.001 * |
| Averaged BMI change (kg/m^2^) | 2505 | **-**0.186 | <0.001 * | 1861 | **-**0.192 | <0.001 * | 2015 | **-**0.173 | | <0.001 * |
| Weight change (kg) | 3040 | **-**0.227 | <0.001 * | 2200 | **-**0.228 | <0.001 * | 2202 | **-**0.214 | | <0.001 * |
| Averaged weight change (kg) | 2623 | **-**0.215 | <0.001 * | 1930 | **-**0.225 | <0.001 * | 2066 | **-**0.199 | | <0.001 * |
| **Time period T-3 to T0** |  |  |  |  |  |  |  |  | |  |
| HbA1c change (mmol/mol) | 2276 | 0.036 | 0.086 ^#^ | 1643 | 0.051 | 0.040 ^#^ | 1670 | **-**0.114 | | <0.001 * |
| Averaged HbA1c change (mmol/mol) | 1822 | 0.055 | 0.020 ^#^ | 1346 | 0.075 | 0.006 ^#^ | 1489 | **-**0.111 | | <0.001 * |
| BMI change (kg/m^2^) | 2171 | **-**0.157 | <0.001 * | 1609 | **-**0.167 | <0.001 * | 1654 | **-**0.181 | | <0.001 * |
| Averaged BMI change (kg/m^2^) | 1734 | **-**0.178 | <0.001 * | 1315 | **-**0.190 | <0.001 * | 1477 | **-**0.180 | <0.001 * | |
| Weight change (kg) | 2307 | **-**0.161 | <0.001 * | 1680 | **-**0.178 | <0.001 * | 1724 | **-**0.219 | <0.001 * | |
| Averaged weight change (kg) | 1840 | **-**0.202 | <0.001 * | 1367 | **-**0.237 | <0.001 * | 1527 | **-**0.218 | <0.001 * | |

*, The variable in percentage revealed a similar significant association with weight change T0 – T+1 in kg (data not shown).

^#^, The variable in percentage revealed a non-significant association with weight change T0 – T+1 in kg (data not shown).

*Complete insulin group* Results of the various pre-insulin HbA1c change measures appeared to vary. Concerning the time periods T-1 to T0, T-2 to T0 and T-3 to T0, all measures of pre-insulin weight change and BMI change were inversely associated with weight change. Based on both the correlation coefficient and available data (r=-0.227 with p<0.001, for n=3040), pre-insulin weight change (kg) in time period T-2 to T0 was chosen as main covariate and visualized by figure S3.

*Matched groups* Similar to the insulin subgroup concerning the time periods T-1 to T0, T-2 to T0 and T-3 all measures of pre-insulin weight change and BMI change were inversely associated with weight change. In contrast to the insulin subgroup, an inverse association of baseline HbA1c with weight change was found (r=-0.113 with p<0.001). Concerning the time periods T-1 to T0, T-2 to T0 and T-3 to T0, all measures of pre-insulin HbA1c change were inversely associated with weight change.

| 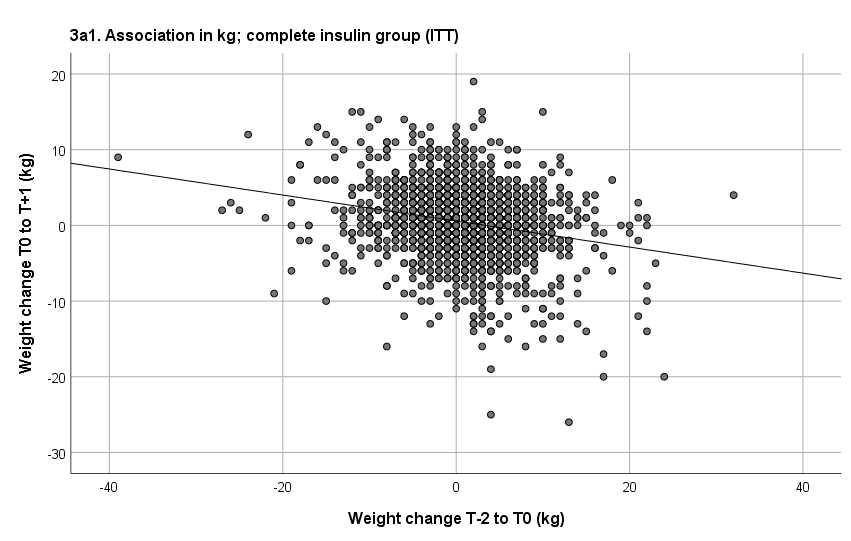 | 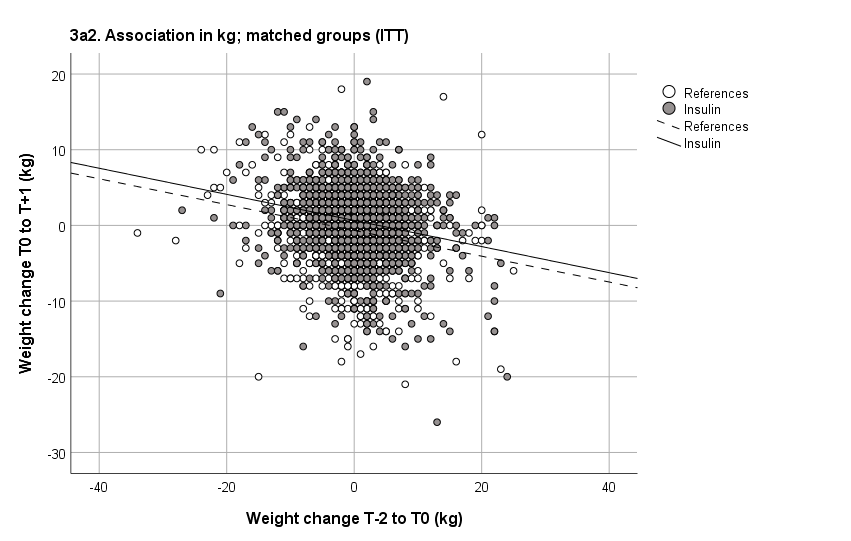 |
| --- | --- |
| 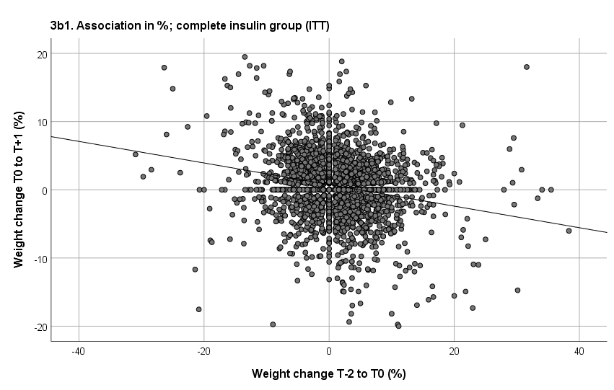 | 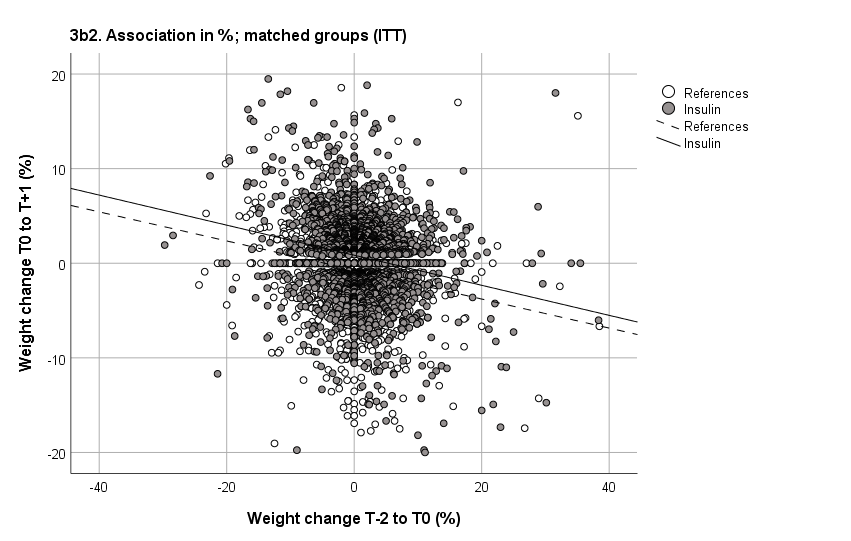 |

**Figure S3.** Association of weight change in time period T-2 to T0 (x-axis) with weight change in time period T0 to T+1 (y-axis) in both kg and %, for both the complete insulin group and the matched groups.

**Table S5.** Comparison of patients, concerning the ITT-matched analysis set.

|  | **Insulin subgroup** | | | **Matched references** | | **Difference** |
| --- | --- | --- | --- | --- | --- | --- |
|  | n | Summary statistics | n | | Summary statistics |  |
| Age (years) | 2812 | 68.1 (±10.1) | 2812 | | 68.1 (±10.1) | 0 |
| Sex (men) | 2812 | 1389 (49.4%) | 2812 | | 1389 (49.4%) | 0% |
| Age at diagnosis (years) | 2812 | 59.7 (±9.8) | 2812 | | 59.8 (±9.7) | -0.1 |
| Diabetes duration (years) | 2812 | 8.3 (±4.1) | 2812 | | 8.2 (±4.1) | 0.1 |
| Main treatment group   - Diet - OGLD - OGLD and insulin - Insulin only | 2812 | 0 (0.0%)  0 (0.0%)  2067 (73.5%)  745 (26.5%) | 2812 | | 1087 (38.7%)  1725 (61.3%)  0 (0.0%)  0 (0.0%) | NA |
| Insulin types (not exclusive)   - Short-acting - Premixed - Basal - NPH | 2812  2812  2812  2812 | 331 (11.8%)  883 (31.4%)  1245 (44.3%)  606 (21.6%) | 0 | | NA | NA |
| OGLD types (not exclusive)   - - Metformin   - Sulfonylureas   - Thiazolidinediones   - Repaglinide   - DDP4 inhibitors   - GLP-1 receptor agonists | 2812  2812  2812  2812  2812  2812 | 1800 (64.0%)  1024 (36.4%)  26 (0.9%)  1 (0.0%)  30 (1.1%)  1 (0.0%) | 2812  2812  2812  2812  2812  2812 | | 1436 (51.1%)  945 (33.6%)  84 (3.0%)  1 (0.0%)  39 (1.4%)  4 (0.1%) | 12.9%  2.8%  -2.1%  0.0%  -0.3%  -0.1% |
| Metformin use change T0 to T+1   - Stable no use - Decrease (yes to no) - Increase (no to yes) - Stable continued use | 2812 | 797 (44.9%)  180 (10.1%)  149 (8.4%)  651 (36.6%) | 2812 | | 715 (39.9%)  209 (11.7%)  191 (10.7%)  675 (37.7%) | 5%  -1.6%  -2.3%  -1.1% |
| Sulfonylurea use change T0 to T+1   - Stable no use - Decrease (yes to no) - Increase (no to yes) - Stable continued use | 2812 | 1715 (61.0%)  266 (9.5%)  73 (2.6%)  758 (27.0%) | 2812 | | 1687 (60.0%)  157 (5.6%)  180 (6.4%)  788 (28.0%) | 1%  3.9%  3.8%  -1.0% |
| Lipid lowering drugs | 2812 | 1822 (64.8%) | 2812 | | 1489 (53.0%) | 11.8% |
| Lipid lowering drug use change T0 to T+1   - Stable no use - Decrease (yes to no) - Increase (no to yes) - Stable continued use | 2812 | 803 (28.6%)  299 (10.6%)  187 (6.7%)  1523 (54.2%) | 2812 | | 1096 (39.0%)  214 (7.6%)  227 (8.1%)  1275 (45.3%) | -10.4%  3.0% -1.4%  8.9% |
| Diuretics | 2812 | 920 (32.7%) | 2812 | | 752 (26.7%) | 6.0% |
| Diuretic use change T0 to T+1   - Stable no use - Decrease (yes to no) - Increase (no to yes) - Stable continued use | 2812 | 1746 (62.1%)  173 (6.2%)  147 (5.2%)  747 (26.6%) | 2812 | | 1912 (68.0%)  128 (4.6%)  148 (5.3%)  624 (22.2%) | -5.9%  1.6%  -0.1%  4.4% |
| Height (m) | 2805 | 170.6 (±9.9) | 2782 | | 170.0 (±9.7) | 0.6 |
| Weight (kg) | 2812 | 86.9 (±15.0) | 2812 | | 86.3 (±14.8) | 0.6 |
| BMI (kg/m^2^) | 2812 | 29.8 (±4.3) | 2812 | | 29.8 (±4.3) | 0.0 |
| SBP (mmHg) | 2804 | 138.9 (±17.0) | 2794 | | 139.5 (±17.1) | -0.6 |
| DBP (mmHg) | 2790 | 77.7 (±10.2) | 2781 | | 78.4 (±9.5) | -0.7 |
| HbA1c (mmol/mol) | 2771 | 57 (±10.6) | 2722 | | 48.3 (±7.7) | 8.7 |
| HbA1c change T0 to T+1 (mmol/mol) | 2747 | -0.74 (±10.0) | 2696 | | 0.34 (±6.9) | -1.1 |
| HbA1c T+1 | 2785 | 56.7 (±10.0) | 2793 | | 48.6 (±8.1) | 8.1 |
| Total cholesterol (mmol/L) | 2736 | 4.3 (±1.0) | 2715 | | 4.5 (±1.0) | -0.2 |
| HDL-cholesterol (mmol/L) | 2726 | 1.2 (±0.4) | 2707 | | 1.3 (±0.4) | -0.1 |
| Cholesterol/ HDL ratio (mmol/L) | 2735 | 3.8 (±1.2) | 2712 | | 3.7 (±1.1) | 0.1 |
| LDL-cholesterol (mmol/L) | 2663 | 2.3 (±0.8) | 2651 | | 2.4 (±0.8) | -0.1 |
| Triglycerides (mmol/L) | 2703 | 1.6 (1.1 – 2.2) | 2684 | | 1.5 (1.1 – 2.1) | 0.1 |
| Serum creatinine (umol/L) | 2729 | 81.1 (±26.6) | 2699 | | 79.8 (±24.0) | 1.3 |
| Smoking (yes) | 2769 | 597 (21.6%) | 2766 | | 553 (20.0%) | 1.6% |
| Alcohol (yes) | 1434 | 267 (18.6%) | 1429 | | 329 (23.0%) | -4.4% |
| Alcohol change T0 to T+1   - Stable no use - Decrease (yes to no) - Increase (no to yes) - Stable continued use | 1021 | 793 (77.7%)  40 (3.9%)  40 (3.9%)  148 (14.5%) | 1009 | | 744 (73.7%)  42 (4.2%)  43 (4.3%)  180 (17.8%) | 4%  -0.3%  -0.4%  -3.3% |
| Physical activity (adequate) | 2236 | 1005 (44.9%) | 2231 | | 1098 (49.2%) | -4.5 |
| Physical activity change T0 to T+1   - Stable not adequate - Decrease (adequate to not adequate) - Increase (not adequate to adequate) - Stable adequate | 1777 | 797 (44.9%)  180 (10.1%)  149 (8.4%)  651 (36.6%) | 1790 | | 715 (39.9%)  209 (11.7%)  191 (10.7%)  675 (37.7%) | 5%  -1.6%  -2.3%  -1.1% |

**Table S6.** Univariate regression analysis concerning the ITT-matched analysis set.

|  | **Matched groups combined** | | | | **Insulin subgroup** | | | | **Matched references** | | | |
| --- | --- | --- | --- | --- | --- | --- | --- | --- | --- | --- | --- | --- |
|  | **n** | **B (SE)** | **p-value** | **R^2^** | **n** | **B (SE)** | **p-value** | **R^2^** | **n** | **B (SE)** | **p-value** | **R^2^** |
|  |  |  |  |  |  |  |  |  |  |  |  |  |
| **Pre-insulin** |  |  |  |  |  |  |  |  |  |  |  |  |
| Weight change T-2 to T0 (kg) | 3594 | -0.160 (0.013) | <0.001 | 0.042 | 1816 | -0.175 (0.017) | <0.001 | 0.053 | 1778 | -0.175 (0.018) | <0.001 | 0.049 |
| **Baseline/ T0** |  |  |  |  |  |  |  |  |  |  |  |  |
| Weight (kg) | 5624 | -0.018 (0.003) | <0.001 | 0.005 | 2812 | -0.022 (0.005) | <0.001 | 0.008 | 2812 | -0.014 (0.005) | 0.002 | 0.003 |
| HbA1c (mmol/mol) | 5493 | 0.014 (0.005) | 0.005 | 0.001 | 2771 | 0.019 (0.007) | 0.007 | 0.003 | 2722 | -0.055 (0.009) | <0.001 | 0.013 |
| Metformin | 5624 | -0.162 (0.103) | 0.115 | 0.000 | 2812 | -0.301 (0.151) | 0.046 | 0.001 | 2812 | -0.268 (0.141) | 0.057 | 0.001 |
| Sulfonylureas | 5624 | -0.026 (0.107) | 0.804 | 0.000 | 2812 | 0.086 (0.150) | 0.569 | 0.000 | 2812 | -0.199 (0.149) | 0.183 | 0.001 |
| Lipid lowering drugs | 5624 | 0.041 (0.103) | 0.690 | 0.000 | 2812 | 0.038 (0.151) | 0.803 | 0.000 | 2812 | -0.165 (0.141) | 0.244 | 0.000 |
| Diuretics | 5624 | -0.177 (0111) | 0.112 | 0.000 | 2812 | -0.135 (0.154) | 0.381 | 0.000 | 2812 | -0.360 (0.159) | 0.024 | 0.002 |
| Alcohol | 2863 | 0.199 (0.173) | 0.249 | 0.000 | 1434 | 0.377 (0.255) | 0.140 | 0.002 | 1429 | 0.143 (0.233) | 0.541 | 0.000 |
| Physical activity (adequate) | 4467 | 0.258 (0.113) | 0.022 | 0.001 | 2236 | 0.201 (0.159) | 0.207 | 0.001 | 2231 | 0.385 (0.158) | 0.015 | 0.003 |
| **T0 to T+1** |  |  |  |  |  |  |  |  |  |  |  |  |
| HbA1c change T0 to T+1 (mmol/mol) | 5443 | 0.041 (0.006) | <0.001 | 0.008 | 2747 | 0.013 (0.007) | 0.077 | 0.001 | 2696 | 0.111 (0.010) | <0.001 | 0.041 |
| Metformin stopped T0 to T+1 | 5624 | -0.268 (0.180) | 0.135 | 0.000 | 2812 | -0.101 (0.246) | 0.681 | 0.000 | 2812 | -0.553 (0.259) | 0.033 | 0.002 |
| Metformin initiated T0 to T+1 | 5624 | -0.271 (0.201) | 0.177 | 0.000 | 2812 | 0.005 (0.321) | 0.987 | 0.000 | 2812 | -0.284 (0.255) | 0.266 | 0.000 |
| Sulfonylurea use stopped T0 to T+1 | 5624 | -0.312 (0.193) | 0.105 | 0.000 | 2812 | -0.136 (0.247) | 0.581 | 0.000 | 2812 | -0.928 (0.307) | 0.003 | 0.003 |
| Sulfonylurea use initiated T0 to T+1 | 5624 | -0.225 (0.245) | 0.359 | 0.000 | 2812 | -0.011 (0.455) | 0.981 | 0.000 | 2812 | -0.037 (0.288) | 0.897 | 0.000 |
| Lipid lowering drugs stopped T0 to T+1 | 5624 | 0.047 (0.177) | 0.790 | 0.000 | 2812 | 0.058 (0.235) | 0.804 | 0.000 | 2812 | -0.158 (0.266) | 0.553 | 0.000 |
| Lipid lowering drugs initiated T0 to T+1 | 5624 | 0.295 (0.195) | 0.130 | 0.000 | 2812 | 0.252 (0.290) | 0.385 | 0.000 | 2812 | 0.415 (0.259) | 0.108 | 0.001 |
| Diuretics stopped T0 to T+1 | 5624 | -0.471 (0.226) | 0.037 | 0.001 | 2812 | -0.320 (0.301) | 0.287 | 0.000 | 2812 | -0.834 (0.338) | 0.014 | 0.002 |
| Diuretics initiated T0 to T+1 | 5624 | -0.492 (0.228) | 0.031 | 0.001 | 2812 | -0.078 (0.325) | 0.810 | 0.000 | 2812 | -0.901 (0.315) | 0.004 | 0.003 |
| Physical activity decrease T0 to T+1 | 3567 | 0.152 (0.202) | 0.453 | 0.000 | 1777 | -0.063 (0.293) | 0.829 | 0.000 | 1790 | 0.405 (0.275) | 0.141 | 0.001 |
| Physical activity increase T0 to T+1 | 3567 | -0.457 (0.214) | 0.033 | 0.001 | 1777 | -0.548 (0.319) | 0.086 | 0.002 | 1790 | -0.283 (0.286) | 0.323 | 0.001 |
| **T+1** |  |  |  |  |  |  |  |  |  |  |  |  |
| HbA1c T+1 (mmol/mol) | 5568 | 0.044 (0.005) | <0.001 | 0.013 | 2785 | 0.033 (0.007) | <0.001 | 0.007 | 2783 | 0.029 (0.009) | 0.001 | 0.004 |

**References supplementary appendix**

1. Watson L, Wilson BP, Alsop J, Kumar S. Weight and glycaemic control in type 2 diabetes: what is the outcome of insulin initiation? Diabetes Obes Metab. 2011 Sep;13(9):823-31.

2. Friedewald WT, Levy RI, Fredrickson DS. Estimation of the concentration of low-density lipoprotein cholesterol in plasma, without use of the preparative ultracentrifuge. Clin Chem. 1972 Jun;18(6):499-502.

3. Balkau B, Home PD, Vincent M, Marre M, Freemantle N. Factors associated with weight gain in people with type 2 diabetes starting on insulin. Diabetes Care. 2014 Aug;37(8):2108-13.

4. Yadgar-Yalda R, Colman PG, Fourlanos S, Wentworth JM. Factors associated with insulin-induced weight gain in an Australian type 2 diabetes outpatient clinic. Intern Med J. 2016 Jul;46(7):834-9.
